# Editorial: Brain-heart interactions in stroke

**DOI:** 10.3389/fstro.2025.1586866

**Published:** 2025-03-25

**Authors:** Benjamin Y. Q. Tan, Luigi Sironi, Danesh Soltani, Ching-Hui Sia

**Affiliations:** ^1^Division of Neurology, Department of Medicine, National University Hospital, Singapore, Singapore; ^2^Department of Pharmaceutical Sciences, University of Milan, Milan, Italy; ^3^Cardiac Primary Prevention Research Center (CPPRC), Cardiovascular Diseases Research Institute, Tehran University of Medical Sciences, Tehran, Iran; ^4^Department of Cardiology, National University Heart Centre, Singapore, Singapore

**Keywords:** brain-heart axis, heart, brain, stroke, genetics, intracranial atherosclerosis, vascular medicine

Stroke is an important public health priority and is one of the leading causes of death and disability globally (GBD 2021 Stroke Risk Factor Collaborators, 2024). Increasingly, recent research has emphasized the intricate relationships between the brain and heart, revealing how cardiovascular and cerebrovascular health are interconnected in ways that influence both stroke risk and recovery (Ho et al., 2024; Méloux et al., 2020). The studies presented in this Research Topic bring to light critical insights into how various cardiovascular and cerebrovascular conditions—ranging from intracranial atherosclerotic stenosis (ICAS) to atrial fibrillation (AF) and infective endocarditis—offer new directions for diagnosis, prevention, and treatment. These articles converge on a broader, integrated view of stroke, highlighting how the interplay between brain and heart can significantly affect stroke care.

At the heart of this integration is the recognition that stroke is rarely an isolated event but rather part of a broader systemic process that links cerebrovascular health with cardiovascular dysfunction. One of the key insights emerging from the research is the importance of considering vascular health across different territories—such as intracranial and coronary arteries—through a *panvascular medicine* approach. This concept, as explored by Tang et al. underscores the idea that atherosclerosis in one vascular system (e.g., coronary arteries) often signals potential issues in others (e.g., intracranial arteries). By bridging the gap between coronary artery disease and ICAS, researchers are calling for more comprehensive diagnostic methods that do not treat cerebrovascular health in isolation but recognize its interconnectedness with broader vascular disease processes. This approach suggests that understanding and treating atherosclerosis as a systemic issue and learning from previous lessons in cardiovascular disease could improve outcomes for stroke patients by offering more precise diagnoses and targeted interventions that address ICAS.

The relationship between the heart and stroke extends beyond shared vascular mechanisms, with genetic factors playing a critical role in stroke risk. Xu et al. leverage Mendelian Randomization to explore the genetic connections between atrial fibrillation (AF) and other organ systems, including the digestive system. Their study reveals a genetic link between AF and increased susceptibility to cancers of the colon, esophagus, and small intestine. This approach, which uses genetic variants as proxies to assess causality, highlights how genetic predispositions to AF could indirectly elevate the risk of digestive cancers. These findings suggest that AF, though primarily a heart condition, may have systemic effects mediated through ischemic stroke or heart failure, extending its impact to the digestive tract. This research underscores the complex genetic interconnections between cardiovascular diseases and other systemic conditions, highlighting how genetic predispositions can influence the risk of diseases far beyond the heart itself.

The interconnectedness between the brain and heart presents a unique opportunity to enhance stroke management strategies. Yuan et al. illustrate how advanced predictive modelling techniques through the use of Bayesian networks can significantly improve stroke risk prediction in high-risk populations, like infective endocarditis. By incorporating a range of clinical factors—such as hyperlipidemia, hypertension, age, vegetation size, Staphylococcus aureus infection, and early prosthetic valve infective endocarditis—the Bayesian networks model reveals strong associations with IS, offering notable advantages over traditional logistic regression in predicting ischemic stroke in IE patients. This robust model, developed through extensive training and validation, highlights the growing need for sophisticated, personalized medicine in stroke care. This study exemplifies how leveraging brain-heart interactions can not only enhance stroke risk prediction but also improve comprehensive care for patients with multi-system conditions like infective endocarditis.

The prediction of recurrent cerebro-cardiovascular events (CCVEs) after ischemic stroke relies heavily on understanding the interplay between various cardiovascular parameters and stroke risk. Filchenko et al. highlight how factors such as blood pressure variability (BPV), heart rate variability (HRV), and endothelial dysfunction, when assessed early post-stroke, can serve as critical predictors of future CCVEs. This research emphasizes the need to monitor cardiovascular health not only during the acute phase of stroke but also over the long term. It underscores the complexity of stroke recovery, where factors like elevated BPV, nocturnal HRV, and endothelial dysfunction significantly influence the risk of subsequent cardiovascular events. By recognizing these cardiovascular markers, clinicians can better stratify patients for future risk, thereby enhancing post-stroke care. This research further strengthens the brain-heart connection, advocating for a more integrated and holistic approach to stroke prevention and management.

Taken together, the articles in this Research Topic illustrate that brain-heart interactions are central to understanding stroke pathogenesis and its outcomes. Whether through the shared vascular mechanisms that link intracranial atherosclerosis and coronary artery disease, the genetic and systemic links between heart conditions and cancer, advanced predictive models for infective endocarditis, or the role of cardiovascular markers in post-stroke recurrence, the research showcased emphasizes the need for an integrated, multi-dimensional approach to stroke care. Stroke should not be viewed solely as a brain event but as part of a larger systemic process that involves the heart, vasculature, and other organs. In this regard, the concept of *panvascular medicine* could offer a framework for more comprehensive care, where interventions targeting both the heart and brain are employed to prevent stroke and improve patient outcomes. Similarly, the findings on genetic predispositions, predictive modeling, and cardiovascular markers point toward a future in which personalized, data-driven care can be implemented to manage stroke risk more effectively.

As the body of research on brain-heart interactions continues to grow, it is clear that a more holistic approach to stroke prevention and management is necessary. The research presented here marks an important step forward in this direction, offering new insights into how the brain and heart influence one another and how understanding this complex relationship can ultimately improve stroke prognosis and reduce recurrence. By adopting a more integrated view of stroke care—one that takes into account the full spectrum of cardiovascular and cerebrovascular factors—clinicians can provide more effective, personalized treatment strategies that will help mitigate the global burden of stroke.
